# Idiopathic inflammatory myopathy comorbid with Sturge-Weber syndrome: a case report

**DOI:** 10.1186/s12883-019-1303-9

**Published:** 2019-05-03

**Authors:** Li Deng, Dongmei Wang, Ni Ruan, Ping Fu

**Affiliations:** 1 Department of Rheumatology and Clinical Immunology, The Second Affiliated Hospital of Kunming Medical University, 374 Dianmian Avenue Wuhua District, Kunming, 650101 Yunnan China; 2 Department of Neurology, Nanfang Hospital, Southern Medical University, Guangzhou, 510515 Guangdong China

**Keywords:** Sturge-Weber syndrome (SWS), Idiopathic inflammatory myopathy (IIM)

## Abstract

**Background:**

Sturge-Weber syndrome (SWS) is a rare and sporadic congenital neurocutaneous disorder, that is characterized by facial venous capillary malformation (port-wine birthmark), leptomeningeal venous malformation (angiomatosis), glaucoma, and neurologic problems. SWS can comorbid with other disorders in some patients, however, muscular abnormalities have still not been reported in patients with SWS.

**Case presentation:**

A forty-one-year-old Chinese female who had left side port-wine stain, ipsilateral glaucoma, and hypothyroidism was included in the present study. The neurocutaneous and endocrine symptoms were consistent with the SWS diagnostic criteria. Meanwhile, the patient had progressive weakness on her both arms and legs, dramatically elevated creatine kinase (CK) and myoglobin levels, and elevated antinuclear and anti-Ro52 antibodies. Intravenous methylprednisolone (MP) (80 mg/d), methotrexate, and intravenous cyclophosphamide were administrated and the weakness of the patient was gradually relieved with normal serum CK level.

**Conclusions:**

We reported the first case of SWS comorbid with inflammatory myopathy. The underlying mechanism for SWS and idiopathic inflammatory myopathy is still not clear, and further researches need to be conducted to deeply explore the mentioned mechanism.

## Background

Sturge-Weber syndrome (SWS) is a rare and sporadic congenital neurocutaneous disorder, which is characterized by facial venous capillary malformation (port-wine birthmark), leptomeningeal venous malformation (angiomatosis), and glaucoma and neurologic problems (e.g., seizures, stroke-like episodes, mental retardation, migraine, and hemiparesis) [1]. Recently, a somatic mutation of the guanine nucleotide-binding protein G(q) subunit alpha (GNAQ) gene has been described as a cause of the disease [2]. The clinical presentation and prognosis of SWS is highly variable between patients, ranging from no or minimal neurological signs to severe neurologic deficits with recurrent seizures, hemiparesis, visual field deficit, and attention and learning disability [3].

Idiopathic inflammatory myopathies (IIM), collectively known as myositis, are a heterogeneous group of rare conditions that affect multiple organs apart from muscles [4]. The five most recognized types of IIM include dermatomyositis, immune-mediated necrotizing myopathy, overlap myositis (including antisynthetase syndrome), sporadic inclusion-body myositis, and polymyositis [5]. Patients with IIM usually present with symmetrical and proximal limb weakness which develops over weeks or months [6]. Some extramuscular manifestations including arthralgia, non-erosive arthritis, cardiac involvement, pulmonary and gastrointestinal complications have commonly existed [7–9]. Muscle biopsy is very reliable to facilitate the diagnosis, that is conventional among 85% of the patients [10].

Herein, we presented a case of SWS with clinical diagnosis of IIM, and it is the first case that SWS and IIM are observed together. The patient recovered with steroids and immunosuppressant.

## Case presentation

A forty-one-year old female admitted to our hospital with chief complain of Raynaud’s phenomenon for 2 years, and weakness of four extremities for about 2 months. She had morning stiffness of both hands and the symptoms were relieved after 20–30 min of repeated rubbing. No joint pain, fever, oral ulceration, alopecia and rash were noticed. In addition, 2 months before beginning the admission, the patient felt weakness on her both arms and legs, and the weakness aggravated progressively. Difficulties in squatting or standing up, as well as hair combination were noticed. She also complained about muscle tenderness without any difficulty in breathing or swallowing. She was diagnosed as hypothyroid myopathy in a local hospital and levothyroxine was taken. However, her weakness was not improved, accordingly, she was transferred to our department.

The patient’s past medical history was remarkable for left glaucoma with retinal detachment and impaired visual sight in 2015. Besides, she was diagnosed as SWS since childhood. She was also diagnosed as hypothyroidism, and regular levothyroxine replacement was given for several years as well.

Physical examination revealed that the vital sign is stable. 12 cm × 12 cm red patches were visible on her left side, and the boundary was unclear as well. Thyroid nodules and swelling were not palpated. Breathing sounds in both lungs were clear. Proximal muscle strength of her both arms was in level-4 (the UK Medical Research Council criteria), and strength of both proximal legs was in level-3 as well. The distal strengths of her arms and legs were normal, and muscle tenderness was quite obvious in her both arms and legs. Other neurological examinations were not significant.

Laboratory examinations revealed positive anti-nuclear antibody with titer of 1:320 (particle pattern), positive anti-recombinant RO-52 (+++), positive weakly anti-nucleosome antibody (+), and negative anti-neutrophil cytoplasmic antibodies (ANCA). Level of serum creatine kinase (CK) was 11,183 U/L (normal range: 26–140), and myoglobin was 1916 ng/ml (normal range: 25–58). Other chemical examinations revealed that alanine aminotransferase (ALT), aspartate aminotransferase (AST), and lactate dehydrogenase (LDH) were elevated. Thyroid function was normal with 278 IU/ml (normal range:0–115) of elevated anti- thyroglobulin (TG). Electromyography (EMG) showed typical myopathic findings with predominantly proximal muscle weakness. Cranial magnetic resonance imaging (MRI) showed increased anterior chamber of left eye, skin thickening of left facial and abnormal signals of interior wall of left eye, as well as upper and lower recuts. Intracranial brain parenchyma was normal and magnetic resonance angiography (MRA) was not remarkable as well.

Inflammatory myopathy was diagnosed based on the clinical features and laboratory results. Intravenous methylprednisolone (MP (80 mg/d) was administrated and methotrexate (10 mg/week) was intramuscularly injected. The patient’s muscle strength gradually improved, and CK dramatically decreased to 2800 U/L. After 2 weeks, methylprednisolone was gradually tapered to 40 mg/d. As the CK was quite high, intravenous cyclophosphamide was added with a total amount of 6 g.

The patient was discharged with oral MP tapering and methotrexate (MTX) (7.5 mg/week) for maintenance. She was regularly followed-up at our clinic as well. For about half a year, the muscle strength of her arms and legs returned to normal status, and CK was also within normal range.

## Discussion and conclusions

SWS is rare with estimated frequency between 1:20,000 to 1: 50,000 [11]. The clinical features are facial port-wine stains (PWSs) in the V1 distribution (ophthalmic division) of the trigeminal nerve, ipsilateral glaucoma, vascular eye abnormalities, and intracranial occipital-leptomeningeal angiomata. SWS is a disease which affect multiple systems. It is important for neurologists to be aware of other medical issues including the endocrine, psychiatric, ophthalmologic manifestations which can cause and impact the neurologic status of the patients. Recently, some of the clinical features have been reported [1].

The majority of patients with neurologic involvement have seizures in infancy. Besides, neurological deficits can be gradually acquired over a long time. Cognitive impairments are quite common in patients, however, range greatly from minimal attention or mild learning problem to very severe cognitive impairment. Headache and migraines are commonly reported in SWS [1]. In recent years, SWS comorbid with central hypothyroidism has been reported [12], however, the etiology of central hypothyroidism is not clearly elucidated. The possible cause could be the disturbance of hypothalamic-pituitary axis.

In this case, the patient was with typical facial port-wine stain in the V1 distribution, in which ipsilateral glaucoma confirms the diagnosis of SWS. Hypothyroidism also supports the diagnosis of SWS though a primary thyroid function test was lost, and made it difficult to differentiate the central from secondary hypothyroidism. The patient had no prominent brain involvement, history of seizures, headache, or cognitive impairment. The cranial MRI and MRA revealed no abnormality as well.

An impressive feature of this case was that the patient comorbid with severe muscle weakness, dramatically elevated CK and myopathic EMG changes, and elevated autoantibodies as well. The weakness of muscle was alleviated with steroids and immunosuppressant. The differential diagnosis includes myopathy caused by hypothyroidism and other congenital muscle diseases. As no muscle biopsy was conducted, the clinical course, treatment, and the clinical follow-up result of the patient highly suggest the diagnosis of inflammatory myopathy. The diagnosis was confirmed by 2017 European League Against Rheumatism/ American College of Rheumatology classification criteria. This patient’s score was 7.6 and the classification was definite IIM with 91% probability [13]. To our knowledge, no SWS comorbid with IIM has been reported, and the relationship between SWS and IIM is still not clear.

A case of SWS with episodes of rhabdomyolysis has been reported in a Chinese girl and the pathology confirmed the diagnosis of lipid metabolic myopathy [14]. The suggested mechanism is that GNAQ mutation in somatic cells is closely related to dermatology disease. Central nervous system (CNS), musculoskeletal system, eyes, and teeth are typically involved in neurocutaneous disorders [15]. Although muscular disorders have not been found in SWS [16], in terms of tissue development, SWS and muscles originate from the same germ layer [14].

In our study, it was revealed that the somatic GNAQ mutations may be associated with the pathways of IIM. However, no genetic test for GNAQ or muscle biopsy was conducted because of limited financial budget.

We presented a case of SWS comorbid with inflammatory myopathy. The relationship between SWS and IIM is still unclear, and further research is required. In addition, a clinician should consider IIM when a SWS patient has muscle weakness, which may be misdiagnosed as stroke-like episodes of SWS.

## Abbreviations

CK: Creatine kinase
EMG: Electromyography
GNAQ: Guanine nucleotide-binding protein G(q) subunit alpha
IIM: Idiopathic inflammatory myopathy
MP: Methylprednisolone
MRA: Magnetic resonance angiography
MRI: Magnetic resonance imaging
SWS: Sturge-Weber syndrome
